# Traditional Panel Interview versus Multiple Mini-Interview (MMI) in Medical School Admissions: Does Performance differ by Age, Gender, Urban or Rural, or Socioeconomic Status (Findings from one medical school)

**DOI:** 10.15694/mep.2018.0000272.1

**Published:** 2018-12-04

**Authors:** Wanda Parsons, Janet McHugh, Yanqing Yi

**Affiliations:** 1 Memorial University

**Keywords:** Selection, medical school admissions, MMI, panel interviews, SES, urban/rural, gender

## Abstract

This article was migrated. The article was marked as recommended.

Introduction:

Globally, medical schools are trying to widen access and to increase the diversity of their student body to be more representative of the population and to meet the future heath care needs of society. Selection methods must not disadvantage the applicants from priority groups. In Memorial University’s Faculty of Medicine, rural applicants and applicants from low socioeconomic status are priority groups.

**Methods**:

Since 2013, Memorial University has used a combination of traditional panel interviews and MMIs to interview candidates for medical school. We wondered whether applicants who participate in this medical school interview process perform differently on the MMIs compared to the traditional panel interview process and whether performance differs on either of the two interview processes based on age, sex, origin(urban or rural), or socioeconomic status.

Results:

The mean score on the traditional panel interview was higher than that on the MMI. Females scored higher than males on both the traditional panel interview and the MMI. Applicants aged 22 and younger performed worse on both the traditional panel interview and the MMI than the other age groups. Neighborhood socioeconomic status, and urban/rural living status were not significantly related with applicants’ performance on the traditional panel interview or MMI.

Discussion:

The type of interview is not disadvantaging applicants from Memorial University’s priority areas.

## Introduction

The Faculty of Medicine at Memorial University is committed to social accountability. The medical school is situated on an island in the North Atlantic with distributed learning across the province of Newfoundland and Labrador, and other areas of Canada. A large proportion of the population in the province lives in rural and remote communities. To meet the health care needs of the province, the medical school is committedto the equitable selection of a diverse student body in three priority areas:

• Aboriginal peoples
• Students from rural and remote areas
• Economically disadvantaged students

The interview process is part of an inclusive, holistic approach toadmissions at Memorial University. It takes personal characteristics as well as academic history into consideration in the selection process. Since 2013, this medical school has used a hybrid of the traditional (semi-structured) and MMI (structured) (TaMMI). All applicants are selected for interview based on review of their written application, including academics (GPA and MCAT), personal statement, work experience, extracurricular activities and references. Interviewed applicants participate in eight (ten minute) MMI stations with one assessor at each station, and a 30-minute two-person traditional panel interview.

Structured multiple mini-interviews (MMIs) as an interview process for assessing applicants to medical schools has been used since 2002. McMaster University, which has championed the process in Canada, has shown that candidates who are admitted based on scoring well on the MMI perform better on subsequent licensing exams than those who are admitted to medical school based on other assessment processes ( Eva *et al*, 2012). Studies of the effect of socioeconomic status (SES) on interview scores have yielded mixed results. One study found SES had no influence on panel interview scores ( Lumb, Homer and Miller, 2010).Another study found lower MMI scores for applicants with lower SES ( Jerant *et al*, 2015). A group from Manitoba, Canada reported on a studythat suggested medical school applicants who graduated from rural high schools fare worse than candidates from urban high schools on the MMI, despite comparable GPA scores ( Raghavan *et al*, 2013). At the University of Iowa, a team compared interrater reliability and test-retest reliability for both structured and unstructured interviews and found that the unstructured format proved more reliable in both instances ( Axelson *et al*, 2010). Also of note for this current study, a combination of both structured and unstructured interviews proved more reliable than either type alone.

There is also evidence that more (6) stations with a single assessor (interviewer) at each station provided better reliability than fewer (3) stations with two assessors at each station ( Donnon and Paolucci, 2008). As well, interviewer training improves the validity and reliability of the scoring of MMIs ( Roberts *et al*, 2008).

Demographic variables such as ethnicity, gender and SES have been shown to affect performance on cognitive tests ( Jensen, 1980; Suzuki, Short, and Lee, 2011; Davis *et al*, 2013). Some studies show women do significantly better on interview ( Griffin and Hu, 2015; Ross *et al*, 2017)). Other studies have shown older candidates have higher scores ( Jerant *et al*, 2015; Reiter *et al*, 2012). Given Memorial’s need to avoid disadvantaging applicants from priority areas, and findings from previous research, the current study focuses on whether demographic variables, especially SES, and urban/rural origin affect performance on the TaMMI process. Analysis was also done of two other standard variables, age and gender (as self-identified by the applicant). The number of Aboriginal applicants was too small for meaningful statistical analysis.

## Methods

As noted above, the Faculty of Medicine at Memorial University uses a hybrid of the traditional (semi-structured) and MMI (structured) (TaMMI) interview process. Interviewed applicants participate in eight (ten-minute) MMI stations with one assessor at each station, and a 30-minute two person panel traditional interview.

Based on four years of data on the TaMMI process, we present analyses to answer the following research questions:

• Do applicants who participate in the medical school interview process at Memorial University perform differently on the MMIs compared to the traditional interview process?
• Does performance on either of the two interview processes differ based on age, gender, origin (urban or rural), or socioeconomic status?

Automated Geographic Coding Based on the Statistics Canada Postal Code Conversion Files was used to determine SES.( Wilkins and Peters, 2012). Statistics Canada definitions were used to determine rural and urban origin. An urban area was defined as Census Metropolitan Area (CMA) or Census Agglomeration (CA) with more than 50,000 people (including the 50% metropolitan influenced zone surrounding communities). Other areas were defined as rural.

## Results/Analysis

### Pearson correlation

The mean score on traditional interview is significantly higher than that of MMI scores (p-value)

The Pearson correlation between the score on traditional interview and the MMI score is 0.311. This correlation is significantly not zero (p-value)

### Multiple regression results

**MMI score:** Gender and age group are significant when adjusted for urban/rural origin and neighbourhood SES.

• Gender: 0.0041 (p-value)
• Age group:

Notes:

1. Females performed better than males.
2. Compared with the group of ages 27 and above, the young group of ages 22 and below performed worse and the group of ages 23-26 performed not significantly differently.
3. Urban/rural origins and neighborhood SES are not significant. The p-values are as follows:

Urban/rural: 0.1963

Neighborhood SES: 0.1841

**Traditional interview score**: Gender and age group are significant when adjusted for urban/rural origin and neighbourhood SES.

• Gender:
• Age group: 0.0352 (p-value)

Notes:

1. Females performed better than males.
2. Compared with the group of ages 27 and above, the young group of ages 22 and below performed worse and the group of ages 23-26 performed not significantly differently.
3. Urban/rural root and neighborhood SES are insignificant. The p-values are as follows:

Urban/rural: 0.0787

Neighborhood SES: 0.4501

**Table 1.** Demographics and scores for all applicants

	Characteristic	Traditional Score	p-value	MMI Score	p-value
Age group <=22 23 ~ 26 >=27	467 (61.8%) 223 (29.5%) 66 (8.7%)	61.2 *±*10.4 62.8 *±*9.7 63.7 *±*11.4	0.054	51.3 *±*8.8 54.2 *±*8.8 54.8 *±*8.8	<0.0001
Gender Female Male	428 (56.6%) 328 (43.4%)	63.6 *±*10.0 59.6 *±*10.3	<0.0001	53.2 *±*9.0 51.6 *±*8.7	0.013
Urban/rural Urban Rural	584 (77.4%) 171 (22.7%)	61.6 *±*10.0 63.1 *±*9.9	0.087	52.7 *±*8.9 51.7 *±*9.0	0.198
Neighborhood SES Highest Second highest Middle Second lowest Lowest	304 (40.3%) 159 (21.1%) 101 (13.4%) 84 (11.1%) 107 (14.2%)	62.1 *±*10.5 62.1 *±*10.2 61.4 *±*10.6 60.0 *±*10.9 63.1 *±*9.3	0.328	53.2 *±*8.7 52.7 *±*9.1 50.7 *±*9.0 51.6 *±*10.1 52.5 *±*8.1	0.139
Application year 2013 2014 2015 2016	229 (30.3%) 190 (25.1%) 170 (22.5%) 167 (22.1%)	62.2 *±*10.3 62.1 *±*9.3 61.8 *±*11.0 61.3 *±*10.7	0.849	52.6 *±*8.6 53.3 *±*9.0 52.7 *±*8.6 51.2 *±*9.6	0.182

The scores are reported as mean *±* standard deviation.

## Discussion

The mean score on the traditional panel interview is higher than that on the MMI.

Females scored higher than males on both the traditional panel interview and the MMI. This has been shown in other interview domains where females were found to have more effective coping orientations for interviews than did males ( Feeney, McCarthy and Goffin, 2015). Applicants aged 22 and younger performed worse on the MMI and traditional panel interview than the other age groups.

Neighborhood socioeconomic status, and urban/rural origin were not significantly related with applicants’ performance on the traditional panel interview or MMI.

## Conclusion

These results are important as we attempt to widen access to medical school since they show SES and origin (urban /rural) do not affect performance on the MMI and traditional panel interview, suggesting that the interview process is not disadvantaging these applicants.

## Take Home Messages

• SES and origin (urban /rural) do not affect performance on the MMI and traditional panel interview.
• The interview process is not disadvantaging these applicants.
• Applicants score higher on traditional panel interviews than MMI.
• Females perform better on both traditional panel interviews and MMI interviews.
• Younger applicants score lower on both traditional panel interviews and MMI interviews.

## Notes On Contributors

Dr. Wanda Parsons is Assistant Dean for Admissions and Associate Professor of Family Medicine, Faculty of Medicine, Memorial University.

Ms. Janet McHugh is Past Admissions Officer, Faculty of Medicine, Memorial University.

Dr. Yanqing Yi is Associate Professor of Biostatistics, Community Health and Humanities, Faculty of Medicine, Memorial University.
