# Supplementary material for: Traditional Panel Interview versus Multiple Mini-Interview (MMI) in Medical School Admissions: Does Performance differ by Age, Gender, Urban or Rural, or Socioeconomic Status (Findings from one medical school)
Source: MedEdPublish (2016). 2018 Dec 4;7:272. [Version 1] doi: 10.15694/mep.2018.0000272.1 (PMC10711992; doi:10.15694/mep.2018.0000272.1)
Supplement: Peer review history

# Reviewer report: Reviewer response for version 1

**DOI:** 10.21956/mep.19724.r29538

**Published:** 2019-03-14

**Authors:** Richard Hays

**Affiliations:** 1 James Cook University

This review has been migrated. The reviewer awarded 5 stars out of 5 This is a nice study that is important to the institution involved, and probably there as well. It is important that changes in selection processes should not detract from the mission of the school, and that was found. Indeed, selection processes should be designed to reflect the mission. I wonder how much the thinking about attracting students with certain characteristics influenced the design of the MMI? It should have been foremost in the minds of the designers, as selection should be aligned with the mission. Other medical programs should consider conducting similar studies when selection processes are altered.

Reviewer Expertise:

NA

No decision status is available

# Reviewer report: Reviewer response for version 1

**DOI:** 10.21956/mep.19724.r29541

**Published:** 2018-12-13

**Authors:** Balakrishnan Nair

**Affiliations:** 1 Centre for Medical Professional Development and University of Newcastle

This review has been migrated. The reviewer awarded 4 stars out of 5 Medical school selection is major area of research now. Medical schools have societal responsibilities and therefore select students who can meet the societal expectation. However the big question is whether our current selection process is rigorous enough to select the students with those non-cognitive skills to become good doctors to serve the local communities?In this study , from one medical school , they have shown a combination of traditional and MMIs to select students, did not disadvantage students from poor socioeconomic or rural areas. It is good news for all of us who are involved in medical school selection . It will be good to know why they have used both formats of the interviews for the admission process and whether they will abandon the traditional interview which may introduce some bias because of the small sample size of the interviewers

Reviewer Expertise:

NA

No decision status is available

# Reviewer report: Reviewer response for version 1

**DOI:** 10.21956/mep.19724.r29540

**Published:** 2018-12-12

**Authors:** Barbara Griffin

**Affiliations:** 1 Macquarie University

This review has been migrated. The reviewer awarded 2 stars out of 5 This paper adds to the growing evidence that interviews do not discriminate against disadvantaged groups. Unfortunately, information on the number of participants, their ages, gender etc was missing as was information on the scoring system for each type of interview making it difficult to assess what the significant difference in scores between the MMI and traditional interview actually meant. I would be really interested to see if participants changed their rank on one interview compared to the other, i.e., was there a difference in z-scores?

Reviewer Expertise:

NA

No decision status is available

# Reviewer report: Reviewer response for version 1

**DOI:** 10.21956/mep.19724.r29539

**Published:** 2018-12-10

**Authors:** Linda Nield

**Affiliations:** 1 West Virginia University School of Medicine

This review has been migrated. The reviewer awarded 4 stars out of 5 Dr. Parsons and her Admissions Team should be commended for their work in evaluating the fairness of their admissions process. Their brief report answers the question posed in the Title of the work, and a helpful introduction with pertinent references to MMI and traditional interviewing are included for the reader. Although the findings cannot be generalized to all schools' admissions' processes, their work should serve as an impetus for all committees to continually engage in quality improvement.

Reviewer Expertise:

NA

No decision status is available

# Reviewer report: Reviewer response for version 1

**DOI:** 10.21956/mep.19724.r29537

**Published:** 2018-12-05

**Authors:** Tripti Srivastava

**Affiliations:** 1 Datta Meghe Institute of Medical Sciences Deemed University

This review has been migrated. The reviewer awarded 2 stars out of 5 Congratulations to the authors for a novel study. It would have been worthwhile to detail the process of MMT and traditional panel interview and what are their attributes and limitations. The rationale of using both methods should be justified. The data is analysed independently for two methods and records no difference in performance through both methods on the basis of origin and SES. The authors may consider comparison of data to see the co-relation between scores by both methods, pattern of scores and maybe some qualitative data regarding stakeholders perception and preference about the two methods.Lot of scope in this aspect for sure!

Reviewer Expertise:

NA

No decision status is available
